# A Suspected Parasite Spill-Back of Two Novel *Myxidium* spp. (Myxosporea) Causing Disease in Australian Endemic Frogs Found in the Invasive Cane Toad

**DOI:** 10.1371/journal.pone.0018871

**Published:** 2011-04-25

**Authors:** Ashlie Hartigan, Ivan Fiala, Iva Dyková, Miloslav Jirků, Ben Okimoto, Karrie Rose, David N. Phalen, Jan Šlapeta

**Affiliations:** 1 Faculty of Veterinary Science, The University of Sydney, Sydney, New South Wales, Australia; 2 Institute of Parasitology, Biology Centre, Academy of Sciences of the Czech Republic, České Budějovice, Czech Republic; 3 Honolulu Zoo, Honolulu, Oahu, Hawaii, United States of America; 4 Australian Registry of Wildlife Health, Taronga Conservation Society Australia, Mosman, New South Wales, Australia; Duke University Medical Center, United States of America

## Abstract

Infectious diseases are contributing to the decline of endangered amphibians. We identified myxosporean parasites, *Myxidium* spp. (Myxosporea: Myxozoa), in the brain and liver of declining native frogs, the Green and Golden Bell frog (*Litoria aurea*) and the Southern Bell frog (*Litoria raniformis*). We unequivocally identified two *Myxidium* spp. (both generalist) affecting Australian native frogs and the invasive Cane toad (*Bufo marinus*, syn. *Rhinella marina*) and demonstrated their association with disease. Our study tested the identity of *Myxidium* spp. within native frogs and the invasive Cane toad (brought to Australia in 1935, via Hawaii) to resolve the question whether the Cane toad introduced them to Australia. We showed that the Australian brain and liver *Myxidium* spp. differed 9%, 7%, 34% and 37% at the small subunit rDNA, large subunit rDNA, internal transcribed spacers 1 and 2, but were distinct from *Myxidium* cf. *immersum* from Cane toads in Brazil. Plotting minimum within-group distance against maximum intra-group distance confirmed their independent evolutionary trajectory. Transmission electron microscopy revealed that the brain stages localize inside axons. Myxospores were morphologically indistinguishable, therefore genetic characterisation was necessary to recognise these cryptic species. It is unlikely that the Cane toad brought the myxosporean parasites to Australia, because the parasites were not found in 261 Hawaiian Cane toads. Instead, these data support the enemy-release hypothesis predicting that not all parasites are translocated with their hosts and suggest that the Cane toad may have played an important spill-back role in their emergence and facilitated their dissemination. This work emphasizes the importance of accurate species identification of pathogens relevant to wildlife management and disease control. In our case it is paving the road for the spill-back role of the Cane toad and the parasite emergence.

## Introduction

Infectious diseases entering naïve populations are key threatening processes contributing to the precipitous global decline of biodiversity [1]. Currently, more than three quarters of critically endangered species of amphibians are threatened by infectious disease [2]. The chytrid fungus, *Batrachochytrium dendrobatidis* [3], [4], and ranaviruses [5] have been documented as significant drivers of frog population declines. Circumstantial evidence suggests that myxosporean parasites are also causing significant disease in amphibians and may be playing a role in their decline [6], [7], [8], [9]. If myxosporean parasites of amphibians behave in a similar manner to those of fish, then they would have a potential for dissemination outside of their original range and could be pathogenic in naïve populations [7], [10]. The infamous *Myxobolus cerebralis* (Myxosporea) causes a whirling disease in salmonid fish and has been proven to have a devastating impact on wild and farmed fish populations in North America [11]. *Myxobolus cerebralis* was able to spread globally from Europe as the result of the translocations of infected fish and the cosmopolitan distribution of its aquatic invertebrate host – an oligochaete worm *Tubifex tubifex* [10], [12].

Pathological changes associated with *Myxidium* sp. (Myxosporea) were described in livers of green tree frogs (*Litoria caerulea*) and it was suggested that investigations of declining frog populations should consider *Myxidium* spp. as potential pathogens [13]. These parasites were assumed to be *Myxidium immersum* of the invasive Cane toad (*Bufo marinus*, syn. *Rhinella marina*) and thought to have spilled over into a wide range of Australian frogs [14]. Recently, we confirmed the emergence of *Myxidium* parasites in native frogs in Australia, by documenting their earliest occurrence in a specimen from 1966, therefore 31 years after the introduction of the Cane toad in Australia [15]. These findings suggested that genetic characterisation is required to confirm the identity of these parasites in Australian frogs and in Cane toads along their translocation route [16].

The aim of our study was to elucidate the identity of myxosporean parasites in brains and livers of Australian native frogs, the Green and Golden Bell frog (*Litoria aurea*) and the Southern Bell frog (*Litoria raniformis*) of south east Australia [17], and the invasive Cane toad using histological, ultrastructural and genetic characterisation. Because of the apparent identity of these parasites in both native frogs and invasive Cane toads we investigated the possibility that these parasites were translocated into Australian frog populations with the Cane toad. We surveyed Cane toads in Hawaii and compared Australian myxospores with those from the Cane toads in South America. Our data supports a recent emergence of myxosporea in Australian native frogs, however, we found no evidence to suggest that it was introduced to Australia with the Cane toad. Using these data we discuss the mechanisms by which these parasites have dispersed among Australian native and endangered frogs.

## Results and Discussion

### Myxosporean parasites in Australian endangered frogs

A subset of Green and Golden Bell frog tadpoles (*n* = 38) from a semi-captive population in greater Sydney, New South Wales were submitted for necropsy as part of a routine health screening protocol required before animals from this facility could be translocated. At the time it was reported that some tadpoles were exhibiting behavioural changes. Following the preliminary necropsy findings, 10 adult Green and Golden Bell frogs were also submitted for necropsy from this population. Wild caught, adult Southern Bell frogs (*n* = 8) from southern New South Wales that had been held in a captive breeding program were also submitted for diagnostic necropsy as part of an investigation into their weight loss, lethargy, and failure to breed. Both frog species have experienced dramatic population declines across all of their range since the 1980s [18], [19]. Chytridiomycosis has been hypothesised to be a driver in the decline, but cannot account for declines across the entire range of both species as chytrid-free populations have also been lost [19], [20], [21]. Captive breeding programs for these frogs have been established and may play a critical role in saving them from extinction [17], [22].

**Figure 1:**
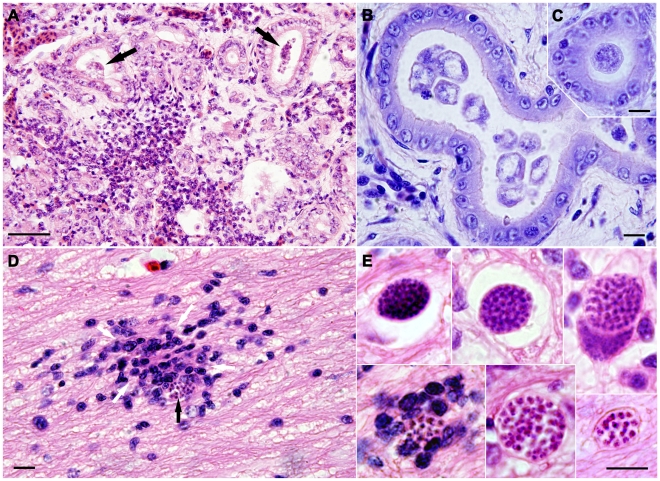
Myxosporea in the Green and Golden Bell frog (*Litoria aurea*) and the Southern Bell frog (*Litoria raniformis*). Affected tadpoles of Green and Golden Bell frog suffered from chronic hepatitic lesions characterised by biliary hyperplasia, hepatitis with lymphoplasmacytic infiltration and fibrosis (A), parasite development was observed inside the bile ducts (arrows). Liver tissue of tadpoles from a semicaptive population of Green and Golden Bell frogs contained early plasmodia found within bile ducts (B, C). Round myxosporean stages (arrow), were identified in the brain and spinal cord and meninges of tadpoles of the Southern Bell frog (D, E). Brain stages were accompanied by a multifocal nonsuppurative meningoencephalitis (D). Each consisted of several secondary cells enclosed in a primary cell wall (E). Scale bars: A - 50 µm; B, C, D, E - 10 µm. A, D, E - H&E, B, C - Giemsa.

Multinucleated, variably-shaped, 5–30 µm in diameter, organisms consistent with myxosporean plasmodia were found within bile ducts of tadpoles of the Green and Golden Bell frog (14/38) (**Figure 1ABC**). Accompanying the organisms were varying degrees of hepatic lesions characterised by biliary duct hyperplasia, moderate to severe loss of peribiliary hepatocytes, hepatitis with lymphoplasmacytic infiltration, and fibrosis (**Figure 1A**). Round and oval myxosporean stages, presumed to be early myxosporean plasmodia, were also identified throughout the central nervous system (CNS; brain and spinal cord) and root ganglia of tadpoles and adults of the Green and Golden Bell frog as well as adults of the Southern Bell frog (**Figure 1DE**). The size of the CNS stages ranged from 5–25 µm in their widest dimension (**Figure 1E**). Each stage in CNS consisted of numerous secondary cells enclosed in a primary cell wall. These brain stages were accompanied by a multifocal nonsuppurative meningoencephalitis found in 7 of 8 adults of the Southern Bell frog (**Figure 1D**). Adults of both Green and Golden Bell frogs (*n* = 1) and Southern Bell frogs (*n* = 7) were found to have plasmodia containing myxospores in the gall bladder. The myxospores morphologically resembled those of the genus *Myxidium*. One Green and Golden Bell tadpole was found to have immature plasmodia in its gall bladder which did not contain mature myxospores. The plasmodia were oval to round, ranged from 0.5–2 mm diameter. We did not identify any myxospores in biliary ducts or other stages either in the liver parenchyma or blood smears from the Green and Golden Bell frog specimens.

Given the apparent ability of *Myxidium* species to cause disease in two species of endangered frog the Australian Registry of Wildlife Health (ARWH) amphibian data base was surveyed for evidence of infection in other species of frogs from New South Wales. Twenty six cases, in nine native frog species, were identified during the period of 1997–2009 that had myxosporean developmental stages either in the brain and nerve ganglia or the bile ducts (**Table S1**). Liver lesions associated with the parasite development were consistent with findings in green tree frogs, *Litoria caerulea* [13]. Lethargy, emaciation or behavioural abnormalities were reported in 26.9% (7/26) of these cases. Given that many of these frogs were examined as part of a pre-translocation health screen, these findings raised a concern that these parasites may be impacting captive breeding colonies and animals released from captive breeding colonies may be serving to disseminate these parasites.

### Myxosporean development confined within myelinated axons of the central nervous system of frogs

**Figure 2:**
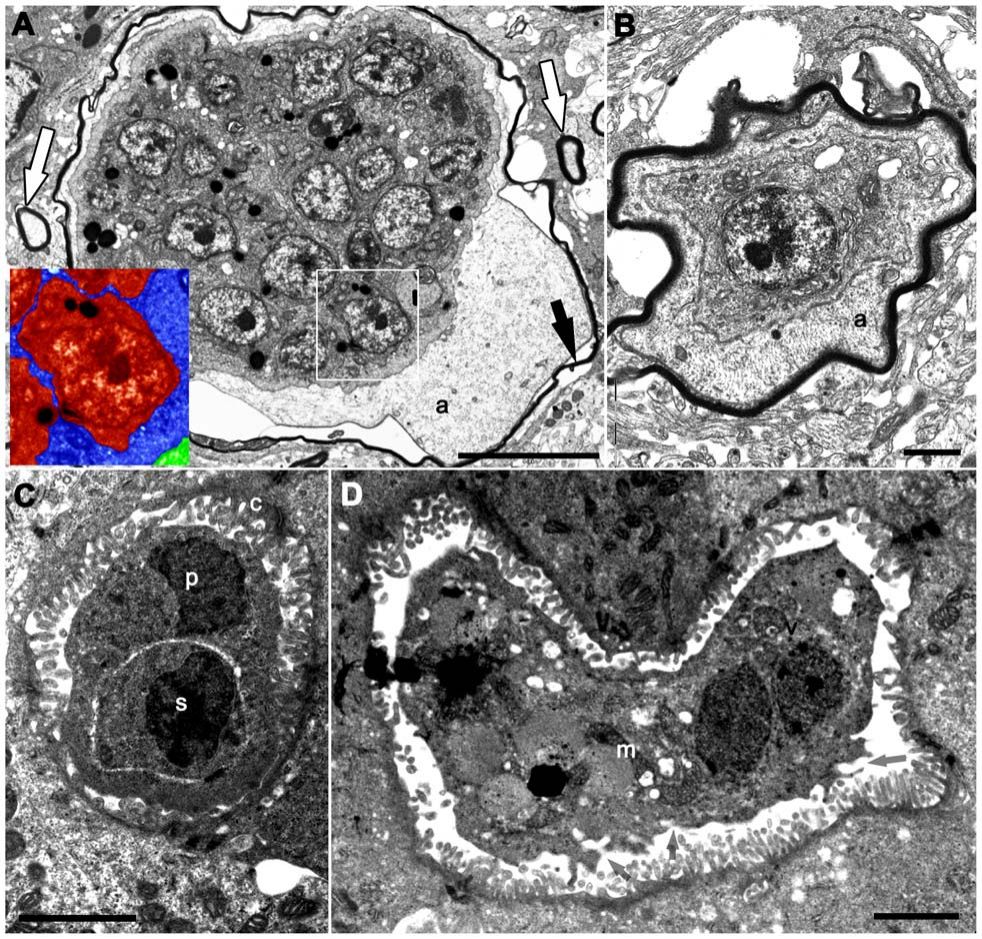
Transmission electron microscopy (TEM) of the developmental stages in the Green and Golden Bell frog tadpoles (*Litoroa aurea*). Brain multinucleated (A) and single nucleated (B) extrasporogonic developmental stages found exclusively within axons (a), enclosed by myelin sheets (black arrows). Developmental stages possessed characteristic cell in cell development consistent with myxosporean extrasporogonic development. The histozoic primary (blue) cells, inside myelinated axon (green), had distinct intracellular cleavages defining the secondary (red) cells (an enlarged area in the white quadrangle; inset A). The parasitised myelinated axon is several times larger in size than the normal myelinated fiber (white arrows). Liver bile duct plasmodia (C) with cell in cell development, primary cell (p) and secondary cell (s). Based on TEM these stages appeared to be unattached to the biliary epithelium (c). The coelozoic plasmodia in bile ducts had numerous mitochondria (m) in the cytoplasm with pinocytic channels (gray arrows) and dispersed lipid inclusions in the cytoplasm (D). Scale bars: A - 1 µm; B - 1 µm; C, D - 2 µm.

Transmission electron microscopy (TEM) of the brain stages revealed extrasporogonic developmental stages exclusively within myelinated axons (black arrows, **Figure 2AB**). Myelinated axons containing these stages were consistently several times larger than the largest normal myelinated fibre (compare the parasitised axon with normal axons - white arrows, **Figure 2A**). These stages possessed characteristic cell in cell development consistent with myxosporean development [23]. They consisted of round primary cells ranging from 5–15 µm in diameter and distinct intracellular cleavages defining the secondary cell development within (blue vs. red area, inset of **Figure 2A**). Extrasporogonic stages were seen to expand to the absolute periphery of the axon membrane in some cases appearing to cause axonal swelling. Axonal lesions may interfere with neurological function and could explain the behaviour changes observed in the Green and Golden Bell frogs.

Myxosporean parasites develop in a variety of tissues, however, intra-axonal development is uncommonly reported and has only been described in a small number of species of fish [24], [25]. Intra-axonal stages resembling those described here were seen in 12 out of 22 spinal cords of the South American toad (*Bufo arenarum*, syn. *Rhinella arenarum*) from Uruguay using TEM, although at the time, the authors were uncertain of the identity of the organisms they were describing [26]. Similarly, parasite stages in the brains of the Cane toad in South America observed under light microscopy and originally interpreted to be *Toxoplasma gondii*, resemble the brain forms described here [27]. Despite the close morphological similarity between the organisms found in the brains of the Australian frogs and the South American toads, it is not possible to determine their relationship because other tissues were not examined (i.e., liver and gallbladder) and they have not been genetically characterised.

The TEMs revealed plasmodia in the early stage of development in the bile ducts of the Green and Golden Bell frog tadpoles. These stages also had cell in cell organisation, with no attachment to the biliary epithelium seen (**Figure 2CD**). The coelozoic plasmodia in bile ducts ranged from 4–25 µm in diameter, had numerous mitochondria, pinocytic channels and dispersed lipid inclusions in the cytoplasm (**Figure 2D**). Compared to the extrasporogonic stages in the brain, the primary cells in the bile ducts had fewer daughter cells. It is suspected that the liver stages were in an earlier stage of development than those found in the brain, because advanced primary cells of myxosporea typically contain large numbers of daughter cells [28].

### Australian native and exotic frogs are parasitised by the same *Myxidium* spp. genotypes

**Figure 3:**
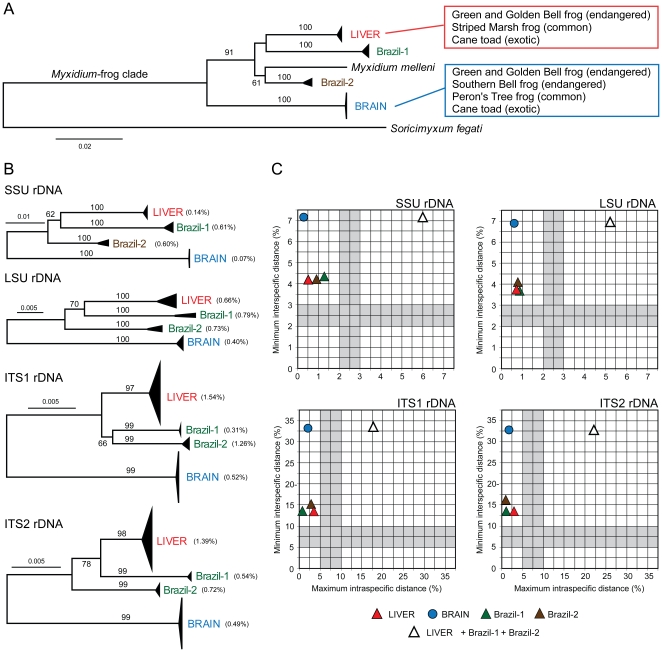
Relationship of genotypes of *Myxidium* spp. in Australian frogs. (A) Phylogenetic tree based on SSU rDNA sequences including the North American *M. melleni* and rooted using a shrew parasite *Soricimyxum fegati*. Parasite hosts and their status in Australia are indicated for the brain and liver genotype. (B) Phylogenetic trees based on SSU rDNA, LSU rDNA, ITS rDNA and ITS2 rDNA with genotype names and mean distances are indicated on the right. (C) The intraspecific and interspecific distance of the brain genotype (blue •), liver (red ▵), Brazil-1 (green ▵), Brazil-2 (brown ▵) and pooled liver+Brazil-1+Brazil-2 genotypes (black outlined ▵) were placed on the graph to evaluate whether they represent candidate species. The graphs are divided into four quadrants that represent different categories of “species” [37]: top left - species concordant with current taxonomy; top right - probable composite species, i.e. candidates for taxonomic split; bottom left - species that have undergone recent divergence, hybridization, or synonymy; bottom right - probable specimen misidentification. Notice that if the liver+Brazil-1+Brazil-2 genotypes (black outlined ▵) are treated as a single species they are resolved in the top right quadrangle suggestive of cryptic species; however when split into the three individual genotypes they became resolved in the top left quadrangle supporting their species status. Trees and distances were inferred using the Minimum Evolution: Maximum Composite Likelihood method in MEGA4 with bootstrap test (1,000 replicates, >50% are shown).

Sequence analysis confirmed the presence of myxosporea in both brain and liver tissues – the liver and the brain genotypes of *Myxidium* spp. (**Figure 3** **, Table S2**). The SSU rDNA sequence amplified from the liver and brain of the Green and Golden Bell frog differed by 9% (855 pairwise alignment length). The sequences obtained from the Southern Bell frog brain and myxospores were identical to each other and matched (100%) the sequence from the brain of the Green and Golden Bell Frog. The rapidly evolving ITS rDNA region spanning across ITS1 rDNA and ITS2 rDNA sequences differed by 34% (375 pairwise alignment length) and 37% (314 pairwise alignment length), respectively. Similarly, more conservative LSU rDNA revealed 7% (2,195 pairwise alignment length) difference between the two sequences. A query of the public repositories of SSU rDNA sequence data using ‘blastn’ returned myxosporean sequences as the most closely related sequences including *Myxidium melleni* (DQ003031) from the gall bladder of the Western Chorus frog (*Pseudacris triseriata triseriata*) in North America [29].

To determine if both genotypes were present in other species of frogs, we collected Striped Marsh frogs (*Limnodynastes peronii*) and Peron's Tree frogs (*Lit. peronii*) from the property where the population of the tadpoles of the Green and Golden Bell frog were raised. The Striped Marsh frog adults had a high prevalence of myxospores and plasmodia (19/22) within their gall bladder and two were found to have the brain stage (2/39). Plasmodia with myxospores were found in bile ducts of 2/38 tadpoles of the Striped Marsh frog and amplified sequences matched the liver genotype. The Peron's Tree frog adults (1/3) were found to possess large numbers of brain extrasporogonic plasmodia as well as myxospores in its gall bladder. Sequencing confirmed identity with the brain genotype of the Green and Golden Bell frog (**Figure 3AB**).

Both genotypes were sequenced from isolated myxospores in gall bladders of adult Cane toads from Lismore and Byron Bay in northern New South Wales (**Figure 3AB**). In Australia, the Cane toad is an introduced species that is still expanding its range with a population confined to the north east corner of the New South Wales [30], [31]. Lismore is the southern most front of the invading toads [32]. Initially we collected gall bladders and found 10% (6/60) and 20% (2/10) Cane toads from Byron Bay positive for myxospores in February and August 2009, respectively. In February 2010, toad sampling revealed 42% (37/82) of toad gall bladders positive for myxospores and presence of extrasporogonic plasmodia in 10% (3/30) of brains (sampled in and in between Byron Bay and Lismore). Brain plasmodia seen in the toads resembled those seen in the Green and Golden Bell frog, the Southern Bell frog and Peron's Tree frog.

**Figure 4:**
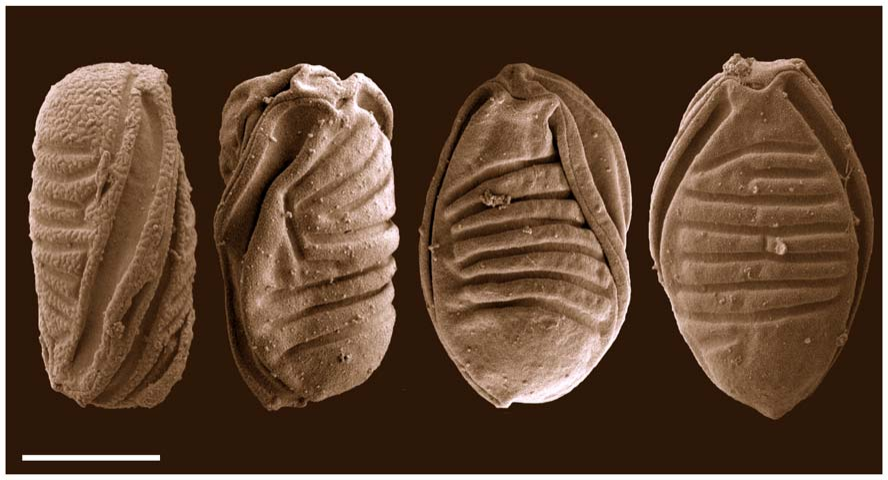
Myxospores of *Myxidium* species - liver genotype. Scanning electron microscopy of myxospores recovered from the gall bladder of the Striped Marsh frog (*Limnodynastes peronii*). Myxospores are ellipsoidal, shell valves have ridges and suture line cross-sectioning the spore. Scale bar, 5 µm.

Using spore morphology, the myxosporea found in Australian frogs belonged to the genus *Myxidium* Bütschli, 1882 (Myxosporea, Myxozoa). All their spores share a characteristic fusiform shape with shell valves either smooth or ridged, suture line cross-sectioning the spore, and two polar capsules that lie one at each end of the spore (**Figure 4**) [23]. *Myxidium*-myxospores recovered from the Green and Golden Bell frog, the Southern Bell frog, the Striped Marsh frogs, the Peron's Tree frog and the introduced Cane toad were of similar size and with overlapping morphological details using light microscopy (**Table S3**). Genotyping revealed that the *Myxidium*-myxospores in the gall bladder of the Striped Marsh frog, but with no detectable brain lesions, were identified as the liver genotype and *Myxidium*-myxospores in the gall bladder of the Southern Bell frog with brain myxosporean development were identified as the brain genotype. Subsequently, the myxospores in the gall bladder of Cane toads were morphologically indistinguishable from those above and sequencing revealed presence of both liver and brain genotypes (**Figure 3AB**). Together with the morphological details of other *Myxidium* spp. of frogs (**Table S3**), our data suggest that the myxospores of divergent amphibian *Myxidium* species have maintained similar structural characteristics and that this shared morphology represents an optimal phenotype that is under strong stabilising selection pressure [33].

### Australian *Myxidium* spp. are not found in Cane toads from Brazil and Hawaii

**Figure 5:**
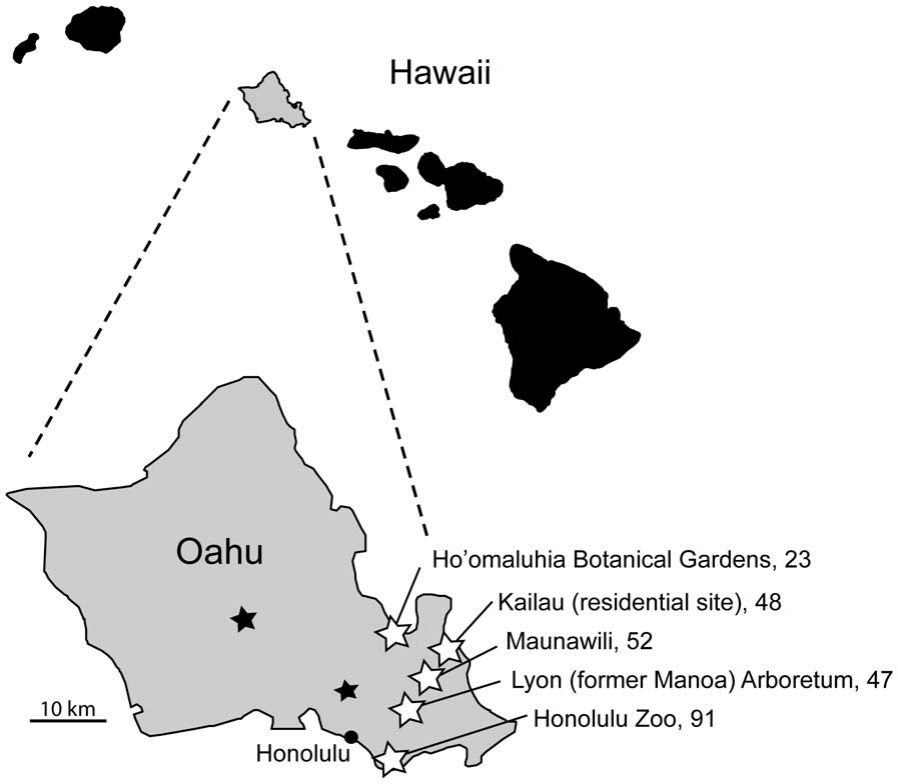
Collection sites of Cane toads (*Bufo marinus*, syn. *Rhinella marina*) surveyed for the presence of *Myxidium* species. Names of the localities are shown together with the number of animals surveyed in February 2010. The Cane toad was introduced (149 individuals) to the Hawaiian island of Oahu from Puerto Rico in April 1932 and introduced to the Manoa Arboretum at the upper end of Manoa Valley and a taro patch adjoining the HSPA Waipio substation and immediately spread throughout the island [34]. In 1935, juvenile and adult toads were collected and in total 101 live individuals arrived in Australia [34]. Two localities recorded as source populations for translocation to Australia are indicated by ★.

The Cane toad was introduced to a large number of countries and states across the world from South America [34]. In Brazil, the Cane toad is the host of *Myxidium immersum* [35]. The parasite was speculated to have been introduced with the Cane toad and to have spread widely in native Australian frogs [14], [36]. Genetic characterisation of *M.* cf. *immersum* myxospores isolated from gall bladders of the Cane toad from Brazil, South America two distinct groups of sequences (Brazil-1 and Brazil-2 genotypes, **Figure 3** **AB**). However, neither of these *M.* cf. *immersum* genotypes matched the *Myxidium* spp. brain or liver genotype from Australian frogs (**Figure 3** **, Table S2**). The Cane toad arrived in Australia from the Hawaiian island of Oahu in 1935 [34]. However, myxosporea were not found in any of the toads (*n* = 261) collected on Oahu in 2010 (**Figure 5**). The probability of observing zero positives in a sample of 261 frogs is 2×10^−6^, using the expected minimum prevalence of 5%. Based on these findings we concluded that the Hawaiian population of Cane toads is free of these myxosporeans and that they were not the source of the myxosporeans that we have identified in Australian frogs and in Cane toads in Australia.

All four DNA markers have resolved identified *Myxidium* spp. genotypes as ‘candidate’ species using barcoding analysis used to unmask cryptic species complexes (**Figure 3C**) [37], [38]. The brain, liver, Brazil-1 and Brazil-2 sequence group appeared in the top left quadrangle representing species that are correctly identified. However, pooling together the liver, Brazil-1 and Brazil-2 sequences suggests that this group consisted of multiple ‘candidate’ species (black outlined ▵, **Figure 3C**). We have arbitrarily applied 2–3% threshold, used in COI of mtDNA barcoding studies [37], [39], for myxosporea more conservative regions (SSU rDNA, LSU rDNA), and suggest using even higher thresholds (5–10%) for more variable regions (ITS1 rDNA, ITS2 rDNA). This diversity observed between these sequence groups exceeds intra-species variation within myxosporean species [12], [40]. The within *Myxidium* spp. group diversity was below 1%, except for the liver sequences at ITS1 rDNA (1.54%) and ITS2 rDNA (1.39%), and for Brazil-2 sequences at ITS1 rDNA (1.26%) (**Figure 3B**). Within a naturally dispersed cosmopolitan parasite such as *Kudoa thyrsites* genetic analysis revealed 0.3% to 7.5% variation of ITS1 rDNA in an individual fish sample [40]. Furthermore, major separations of the marine *K. thyrsites* and the freshwater *Tetracapsuloides bryosalmonae* were correlated with geographic regions and exhibited regional differentiation [40], [41]. The genotyping and morphological analysis of myxospores revealed great diversity of the genus *Ceratomyxa* in fish on the Great Barrier Reef, with 10 new species varying from each other by 1.3% to 28.0% at SSU rDNA [42].

**Figure 6:**
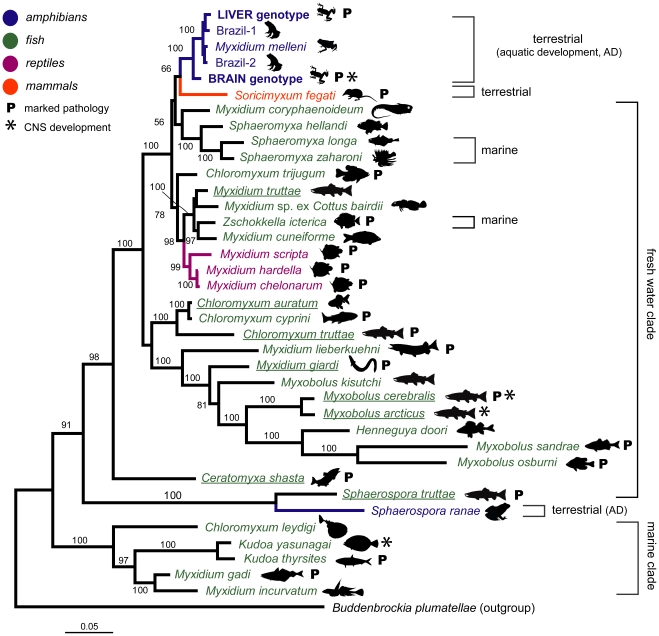
Phylogenetic tree of Myxozoa based on SSU rDNA gene sequence. The Bayesian tree was reconstructed using MrBayes 3.1.2 with a GTR+G+I nucleotide model from the stringent Alignment 3, for details about alignment and phylogenetic reconstruction see Materials and Methods and **Figure S1**. For details on biological traits and GenBank accession numbers see **Table S4**. Bayesian posterior probabilities are shown at the nodes. The myxosporean sequences fall into marine and fresh clades; the exceptions are *Sphaeromyxa* spp. and *Zschokkella icterica* sequences from marine fish that cluster in the fresh water clade [43]. Myxosporean species with documented actinospore development are underlined.

Phylogenetic reconstructions demonstrated monophyly of the liver, brain, Brazil-1, Brazil-2 genotypes and *M. melleni* (**Figure 6** **, Figure S1**). The multiple sequence alignment included a selection of key myxosporean species with attention to select taxa from different host groups and those with different tissue tropisms to map the biology of the frog myxosporean species groups (see Material and methods; **Table S4**). The myxosporean sequences were separated into mostly marine and freshwater species clades [43], [44], [45]. Moreover sequences tended to group according to host groups within the freshwater clade rather than their tissue tropisms (**Figure 6**). Tree reconstruction using distance method and LogDet distance model that takes into account the nonstationary nucleotide frequencies to eliminate artificial grouping of sequences with similar nucleotide frequencies irrespective of true evolutionary relationship revealed trees congruent with the maximum likelihood and Bayesian trees (**Figure S1**). The stringent alignment (Alignment 3) revealed a robust phylogenetic position (ML 99% bootstrap, BP 100% posterior probability and NJ 100% bootstrap) of the brain genotype as a sister group to the monophyletic clade of liver, Brazil-1 and Brazil-2 genotypes, together with *M. melleni*. Interestingly, these frog Myxosporea formed a sister group to the only sequenced myxosporean, *Soricimyxum fegati* (EU232760), from a mammalian host, the Common shrew, *Sorex araneus*. Despite its low support values (ML 52% bootstrap, BP 66% posterior probability and NJ 80% bootstrap) the topology was reconstructed using all methods used (**Figure S1**).

### Conclusion

Accurate species identification is critical for informed wildlife management, as well as having major implications for diagnosis, prevention and control of disease agents in animals and humans [46], [47]. Distinguishing exotic and invasive pathogens from native pathogens requires pre-existing knowledge of their historical distribution – a term ‘cryptogenic’ species has been coined to those species that we can not unambiguously place in either category [48]. This distinction is particularly challenging for *Myxidium* spp. in Australian frogs where no historical baseline data exists and where an invasive host may have carried parasites to new places, because the enemy-release hypothesis predicts that not all parasites are translocated with their hosts [49], [50]. Moreover, *Myxidium* spp. have the extra challenge of finding the invertebrate host necessary for successful establishment in a new environment unless the invertebrate host has been introduced concurrently or is cosmopolitan [10].

To answer the question from where the cryptogenic myxosporean parasites in Australian frogs emerged, we have evaluated whether they are either (i) native or (ii) exotic - they have invaded Australia. Traditional concepts in invasive biology revolve around an invasive species bringing a pathogen along [16], [47], [51], [52]. Assuming this agent of disease is not strictly specific to its original host it spills-over to the local naïve fauna with potentially devastating consequences [51], [52], [53], [54]. An alternative to the spill-over concept is a recently revived theory in invasive ecology called parasite spill-back [55], [56]. The native parasite spill-back requires only an invasive host species that is susceptible to a native parasite. Thus, the potentially serious consequences are not caused by the exotic parasite but the native parasite that is being amplified by the susceptible invasive host species. The majority of parasites in exotic species (67%) are native parasites implying that exotic species commonly have the potential to cause parasite spill-back to native hosts, with effects at both the host individual and population scale [55]. Because, (i) our investigation of Hawaiian Cane toads suggests, that they have cleared myxosporean parasites during their previous translocations, and (ii) the parasites in Brazilian Cane toads are genetically distinct from the gall bladder myxosporea in Australian frogs, we suggest that the most plausible explanation is the parasite spill-back.

The question remains why there were no myxosporean parasites detected prior to the introduction of the Cane toad in 1935 [14], [15]. We offer the following explanation, the parasites were likely present in a low prevalence or have been in a geographically localised area, which may not have been sampled in past frog surveys [15]. The requirement in the parasite spill-back theory is that (i) the invasive host, in our case the Cane toad, acquires and shares a native parasite and (ii) subsequently can successfully disseminate it, due to the high parasite prevalence in the invasive host. Alternatively, if the invasive host does not sufficiently multiply the parasite, the exotic host may serve as a sink for parasites and reduces the overall burden of parasites in the native species due to a dilution effect [57]. Our situation satisfies both conditions of the spill-back theory [55]; (i) the native Australian frogs share the parasites with the invasive Cane toad, and (ii) there is a high prevalence of *Myxidium*-myxospores in the gallbladders of Cane toads in Australia 38% (13/34) from 1980's [14] and up to 42% in our data from 2010. Therefore, we hypothesize that the invasive Cane toad was a susceptible and amplifying host for *Myxidium* spp. under Australian conditions. This hypothesis, however, cannot account for all the data, as both the brain and liver genotype have been detected outside the current Cane toad's range, i.e. greater Sydney and southern New South Wales. Possible explanations for this discrepancy include a second spill-over event where these parasites infected a naïve frog species allowing the parasite to spread beyond the range of the Cane toad. An investigation into the quantitative capacity of each susceptible frog species to produce myxospores will be required before it is possible to start answering this question. Similarly, investigation leading to the elucidation of the *Myxidium* life cycle in frogs will be essential to fully understand the extent of the distribution and risk that these *Myxidium* species pose to the already diminishing frog diversity. This study highlights the insight that can be acquired from studying host-parasite systems disturbed by an invasive host, but it also illustrates the limitations associated with absence of base line data on parasite distributions.

## Materials and Methods

### Samples

#### Ethics statement

Tadpoles and frogs of the Green and Golden Bell frog (*Litoria aurea*), Peron's tree frog (*Litoria peronii*), Striped Marsh frog (*Limnodynastes peronii*) and the Cane toad (*Bufo marinus*, syn. *Rhinella marina*) from New South Wales (NSW), Australia were sampled over a period of two years (2008–2010) under the Department of Environment and Climate Change and Water NSW (Australia) scientific license (S12686 and S12969) and the University of Sydney Animal Ethics Committee approval (N00/9-2008/3/4855 and N00/9-2009/3/5134). All animals in NSW were euthanised with AQUI-S (isoeugenol; AQUI-S Ltd., New Zealand) according to standard protocol [58]. The Cane toad (*B. marinus*) in Hawaii, USA is a pest animal; individuals were euthanised immediately in FINQUEL MS-222 (tricaine methanesulfonate; Argent Laboratories, WA) and isoflurane by the Honolulu Zoo veterinary staff according to a standard humane euthanasia protocol at the Zoo facility (February 2010). The Cane toad (*B. marinus*) material from Brazil was kindly donated by Prof. Ralph Lainson (Departamento de Parasitologia, Instituto Evandro Chagas, Belém, Pará, Brazil) to the laboratory in the Czech Republic (MJ). All animal work was conducted according to relevant national and international guidelines.

#### *Australian native frogs*

Tadpoles and frogs of the Green and Golden Bell frog, Peron's Tree frog, Striped Marsh frog were collected from a semi-captive Green and Golden Bell frog breeding site in greater Sydney, NSW, Australia. This population is actively managed to facilitate increased breeding but individuals of any species can move back and forth between the property and the external environment. Immediately after euthanasia, all animals were dissected and internal organs processed for transmission electron microscopy (liver and brain), histopathology and storage at −20°C. All paraffin blocks were submitted to Australian Registry of Wildlife Health (ARWH, Taronga Conservation Society Australia, Mosman, NSW, Australia). The captive Southern Bell frog adults (*Litoria raniformis*) originally from southern New South Wales, Australia were submitted for health screening through the ARWH; formalin-fixed histological section and tissues in −80°C were retrieved from this study. Gallbladders were removed from all animals and inspected for the presence of myxospores, if present, myxospores were washed in sterile phosphate buffered saline and kept at 4°C.

#### The Australian Registry of Wildlife Health records

We queried the database for all frog entries with a diagnosis of unknown liver and brain ‘protozoan’ infections. Paraffin block and slides were retrieved and inspected for the presence of myxosporean-like stages.

#### Australian invasive Cane toad

Cane toads were sampled from the southern end of their expanding range, Lismore and Byron Bay, New South Wales, Australia. Animals were euthanised and immediately processed for histopathology and their gall bladders inspected for the presence of myxospores as described above.

#### Hawaiian Cane toads

Cane toads from Oahu, Hawaii, US were captured (*n* = 261) by net and hand from areas close to the original release and capture sites according to Easteal [34]. Animals were euthanised in FINQUEL MS-222 and isoflurane by Honolulu Zoo staff according to a standard protocol and gall bladders were examined immediately for the presence of myxospores by wet mount. Portions of liver/gall bladder (*n* = 179) and brain (*n* = 47) were formalin-fixed for histological processing. The probability of observing no positive samples out of the total number of samples collected was calculated using FreeCalc v2; an epidemiological probability calculator demonstrating freedom from disease [59].

#### Brazilian Cane toads

A pooled 70% ethanol fixed sample from 10 individual gall bladders with myxospores of *Myxidium* cf. *immersum* dissected from Cane toads collected in the vicinity of Manaus, Brazil was shipped to and genetically characterised in the Czech Republic.

### Microscopic and histopathological examination

The entire gall bladder contents were examined (wet mount) using 20× and 40× objectives. Myxospores were measured/photographed using a 100× oil-objective BX60 microscope equipped with a DP70 camera (Olympus Australia); a minimum of 30 myxospores from each individual was measured and morphological details evaluated according to Lom and colleagues [28]. Animal tissue or entire individuals were processed for histology; fixed in buffered formalin (10%), processed using standard technique, embedded in paraffin, sectioned and stained with haemotoxylin and eosin (H&E) or Giemsa (Veterinary Histopathology Laboratory, University of Sydney). Individual gall bladders were selected for paraffin embedding, sectioning, Giemsa staining and examined for the presence of myxospores. All sections were observed with an Olympus BX60 microscope (Olympus Australia) equipped with a DP70 camera. All images were acquired as RGB, TIFF files. Images were imported into Creative Suite CS3 (Adobe Systems, San Jose, CA). Adjustment layers for levels and brightness/contrast were used to find the optimal colour balance for investigated features with the original image. All manipulations were made at the original dimensions and at 300 dpi.

### Transmission and scanning electron microscopy

Small portions of brain and liver (<1 mm^3^) were processed for transmission electron microscopy (TEM) immediately after euthanasia; fixed in 2.5% glutaraldehyde in 0.1M sodium (pH 7.0) cacodylate buffer and then postfixed with 2% osmium tetraoxide (OsO_4_), dehydrated through graded ethanols and embedded in Spurr's Resin. The blocks were sectioned, stained and observed at either the Electron Microscopy Unit (University of Sydney) on a Philips CM12 TEM or at the Institute of Parasitology (Academy of Sciences of the Czech Republic) on a JEOL JEM 1010 TEM. Myxospores from Striped Marsh frog's gallbladder were prepared for scanning electron microscopy (SEM); placed on poly-L-lysine coated coverslips, fixed in 1% OsO_4_ in 0.2 M sodium cacodylate buffer (pH 7.0), rinsed in distilled water, dehydrated with a graded acetone series, critical point dried, and coated with gold. Myxospore examination was at either the Electron Microscopy Unit (University of Sydney) using a Philips XL-30 CP SEM or at the Institute of Parasitology (Academy of Sciences of the Czech Republic) on a JEOL JEM 7401F FE-SEM. Both FE-SEM allowed topographic imaging to <1 nm resolution by an efficient in-lens detector. Greyscale images were acquired as TIFF files. Images were imported as greyscale images into Creative Suite CS3 (Adobe Systems, San Jose, CA). Adjustment layers for levels and brightness/contrast were used to find the optimal grey balance for investigated features with the original image in the background. For SEM images the individual myxospores were traced and presented on a black background. All manipulations were made at the original dimensions and at 400 dpi.

### DNA extraction, amplification protocols and sequencing

Tissues for DNA extraction were collected from brain and liver and frozen at −20°C prior to DNA isolation. Total DNA was extracted from 20–40 mg of tissue using the PureLink DNA Kit (Invitrogen Australia, Victoria, Australia) or the DNeasy Blood and Tissue Kit (Qiagen, Victoria, Australia) according to the manufacturers' instructions. DNA from myxospores was extracted using the FastDNA Soil Kit Protocol with a Fast Prep-24 Homogenisation System equipped with QuickPrep Adapter (MP Biomedicals, Australia); the speed setting used was 6.0 for 40 s, otherwise the manufacturer's instructions were followed. Extracted DNA from all samples was resuspended in a final volume of 200 µl (Purelink, DNAeasy) or 50 µl (FastDNA Soil). DNA was quantified and purity assessed using Nanodrop 1000 spectrophotometer (Thermo Scientific, NSW, Australia). Total DNA aliquots were stored at −20°C prior to PCR analysis.

The small subunit rDNA (SSU rDNA) was amplified using MyxoSpecF (5′-TTC TGC CCT ATC AAC TWG TTG-3′) and MyxoSpec R (5′-GGT TTC NCD GRG GGM CCA AC-3′) primer pair [43]. The internal transcribed spacers (ITS) and 5.8S rDNA was amplified using G-ITS-for (5′-GGG ATC CGT TTC CGT AGG TGA ACC TGC-3′) annealing to the 3′-end of SSU rDNA and ITS-Zschok-rev (5′-GAT TCT CAT AGT AAC TGC GAG TG-3′) annealing to the 5′-end of LSU rDNA [60]. The large subunit rDNA (LSU rDNA) was amplified using NLF1050 (5′-AAT CGA ACC ATC TAG TAG CTG G-3′), NLFMyxid (5′-AAT GCT AGG GTT CCR AGT GG-3′), NLR3113 (5′-GTC TAA ACC CAG CTC ACG TTC CCT-3′) and NLR3284 (5′-TTC TGA CTT AGA GGC GTT CAG-3′) in a nested PCR [61]. The expected PCR products of the partial SSU rDNA, complete ITS rDNA and partial LSU rDNA was ∼950 nt, ∼900 nt and ∼2,200 nt, respectively.

PCR amplification mix contained 2×Econo*Taq* PLUS green MasterMix (Lucigen, USA) or 2×MasterMix (Fermentas, USA). Alternatively, PCR was carried with 1 U of *Taq*-Purple polymerase and dNTPs (Top-Bio, Czech Republic). Primers were added at 0.25 µM concentration each. The PCR for SSU rDNA and ITS rDNA was run for 35 cycles (95°C for 0.5 min, 55°C for 1 min, 72°C for 1 min). The nested PCR for LSU rDNA was run for 30 cycles in the first reaction (95°C for 0.5 min, 48°C for 1 min, 72°C for 2 min); for the second PCR an 1 µl aliquot of the first PCR was used as template and run for 35 cycles (95°C for 0.5 min, 52°C for 1 min, 72°C for 1 min). All PCR reactions were initiated at 95°C for 5 min and concluded at 72°C for 7 min. All reactions (25 µl) were run with negative controls (distilled water).

Aliquots of PCR reactions were resolved on a 1% agarose gel, stained with GelRed (Biotium) and visualised using UV-transilluminator. PCR reactions with amplicons of expected mass were either purified using the PCR Micro Kit (Invitrogen Australia, Victoria, Australia) and sent for direct sequencing or cloned using the TA-TOPO for Sequencing (pCR-4) cloning kit (Invitrogen Australia, Victoria, Australia), pGEM-T Easy (Promega) or pDrive (Qiagen) according to the manufacturer's instructions. Plasmids were purified with the MiniPrep Plasmid Kit (Qiagen, Australia).

PCR product and plasmids with target inserts were sequenced using amplification primers, internal primers (LSU rDNA [61]) or primers (T7, SP6, M13R, M13F) within the cloning vector at Supamac (University of Sydney and Prince Albert Molecular Analysis Centre) or Macrogen Inc. (Seoul Korea). Chromatographs were visualised, inspected, assembled, aligned with related sequences and analysed using the CLC Main Workbench 5.5 (CLC bio, Denmark).

### Phylogenetic analyses

To infer SSU rDNA phylogeny of the liver, brain and Brazil genotypes of *Myxidium* spp. we selected taxa based on Fiala [43] and selected additional sequences from additional taxa to select wide Myxosporea (Myxozoa) sampling including diverse groups, hosts and tissue tropism. We used blastn and taxonomy browser at NCBI [62] to select representative sequences and progressively analysed them in MEGA4 [63]; *Buddenbrockia plumatellae* (Myxozoa, Malacosporea) sequence was selected as an outgroup [64], for each taxon formal biological traits have been collected from published sourced (**Table S3**). The final selection of sequences was aligned using Clustal W1.83 [65] using default parameters, followed by manual selection of hypervariable regions and realignment using more relaxed parameters (gap opening: 6, gap extension: 4) and minor adjustment done by eye. The original alignment (Alignment 1) with all 2,711 residues included (1,300 parsimony informative sites) was subject to gblocks 0.91b [66] processing to systematically remove ambiguously aligned positions.

A moderately stringent process (Alignment 2) eliminated variable sites and retaining 1,756 (64% of the original 2,711 positions; 769 parsimony informative sites) and a stringent process (gblocks: Alignment 3) retained 1,206 highly conservative residues (44% of the original 2,711 positions; 446 parsimony informative sites). Alignments 2 and 3 were produced using parameters set to 20 and 30 for the minimum number of sequences for a conserved position as well as for the minimum number of sequences for a flanking position, respectively. For both alignments the minimum length of a block was 10 and gapped positions were included.

All alignments were subject to distance phylogenetic reconstruction in MEGA4 [63], maximum likelihood using PhyML3 [67] and PAUP*4b10 (D.L. Swofford, 2001, PAUP*, Sunderland, MA: Sinauer Associates), and Bayesian using MrBayes3.1.2 [68]. Substitution models for DNA evolution were selected using AIC in ModelTest3.7/ModelTestJ [69]; GTR+G (for original Alignment 1) and GTR+G+I (and moderately stringent Alignment 2 and the stringent Alignment 3). The neighbour joining tree branch robustness was evaluated using bootstrapping method with 1,000 replicates [63]. The maximum likelihood phylogeny tree (PhyML) was generated and its branch robustness assessed by the bootstrapping method with 500 replicates [67]. Maximum likelihood tree (PAUP*) was reconstructed using heuristic search with the NNI swap algorithm with parameters from ModelTest; due to prohibitively long calculation times bootstraps were not calculated. Bayesian support (posterior probability) for the nodes [68] were inferred using two independent runs and four Markov chains and 5,000,000 Markov Chain Monte Carlo (MCMC) steps, discarding the first 2,000,000 steps (40%) as a burn-in. Sampling was performed every 250 generations. Mixing of the chains and convergence was properly checked after runs.

### GenBank Accession numbers submitted with this manuscript

The following sequences were submitted to the GenBank: SSU rDNA HQ822149-HQ822173, LSU rDNA HQ822174-HQ822192 and ITS rDNA HQ822193-HQ822260 from *Myxidium* species of frogs and toads.

## Supporting Information

Table S1 Summary of catalogued cases at the Australian Registry of Wildlife Health (Taronga Conservation Society Australia, Mosman, NSW, Australia) from 1997–2009.
(XLS)

Table S2 Summary of sequence data collected from Myxosporea in frogs.
(XLS)

Table S3 Summary of morphology of myxospores (*Myxidium* spp.) recovered from gall bladders in frogs.
(XLS)

Table S4 Summary of SSU rDNA sequences used to reconstruct myxosporean phylogeny.
(XLS)

Figure S1 Phylogenetic trees inferred using SSU rDNA of Myxosporea. The alignments (Alignment 1–3) are described in Material and Methods. Alignment 1: 2711 positions, 975 constant, 437 variable parsimony-uninformative, 1,300 parsimony-informative. Alignment 2: 1756 (64% of the original 2711 positions), 689 constant, 298 variable parsimony-uninformative, 769 parsimony-informative. Alignment 3: 1206 (44% of the original 2711 positions), 551 constant, 209 variable parsimony-uninformative, 446 parsimony-informative. The trees were reconstructed using PhyML 3.0 (GTR+G+I) [67], PAUP*4b10 (D.L. Swofford, 2001, PAUP*, Sunderland, MA: Sinauer Associates) with models selected using AIC in ModelTest3.7 (Alignment 1: GTR+G, Alignment 2: GTR+G+I, Alignment 3: GTR+G+I) [69], MrBayes3.1.2 (GTR+G+I) [68]. The maximum likelihood phylogeny (PhyML) tree robustness was assessed by the bootstrapping method with 500 replicates [41]. Bayesian support (posterior probability) for assessed (MrBayes) using two independent runs and four Markov chains and 40% steps discarded as a burn-in [68]. All trees were rooted using *Buddenbrockia plumatellae* sequence. The sequence taxa are on the right of the tree (for details see **Table S4**).
(PDF)
