# Supplementary material for: A Suspected Parasite Spill-Back of Two Novel Myxidium spp. (Myxosporea) Causing Disease in Australian Endemic Frogs Found in the Invasive Cane Toad
Source: PLoS One. 2011 Apr 25;6(4):e18871. doi: 10.1371/journal.pone.0018871 (PMC3081827; doi:10.1371/journal.pone.0018871)

SUPPLEMENTARY FIGURE (TREE SSU rDNA - S1)

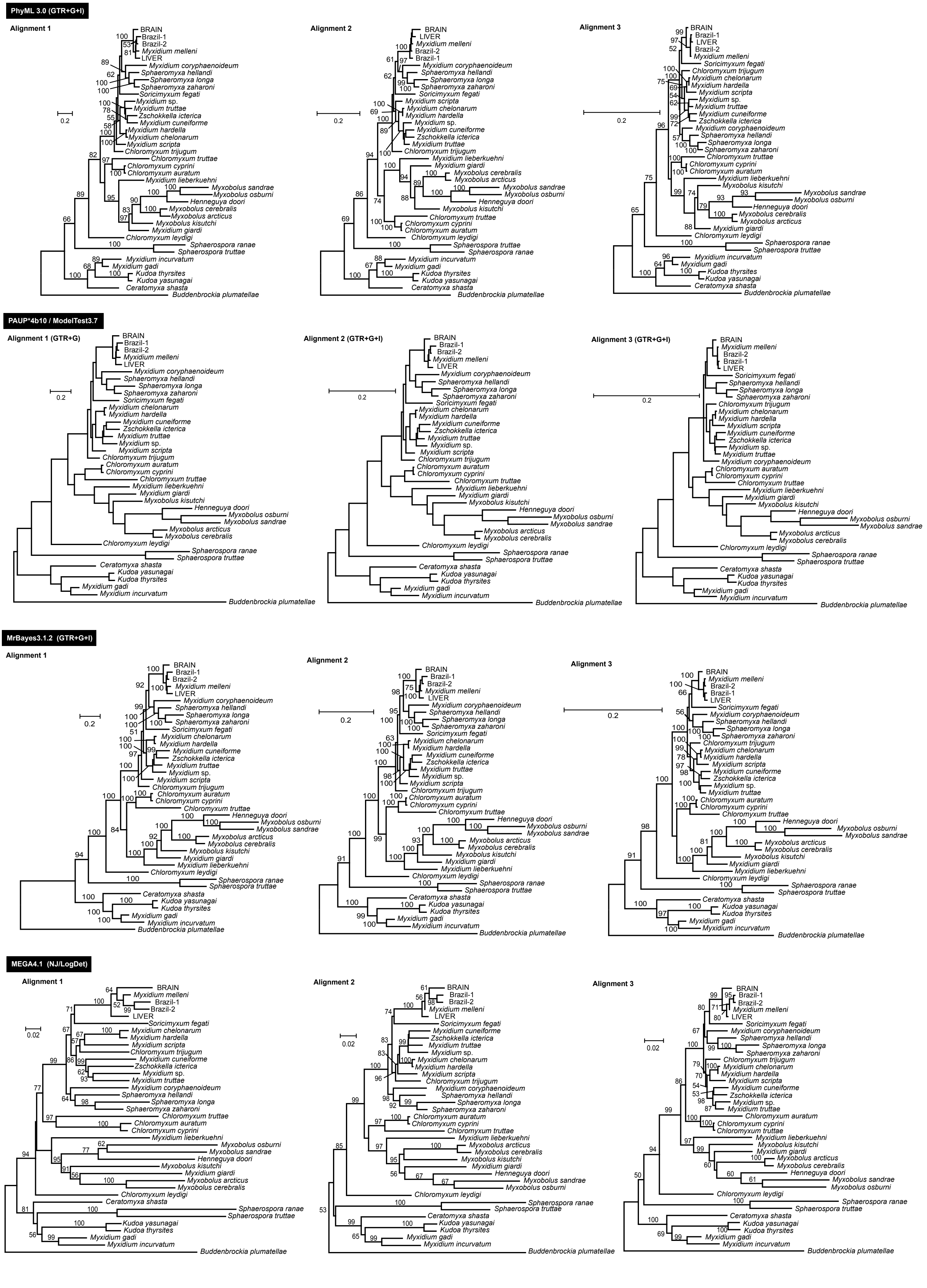

Supplement: Figure S1 — Phylogenetic trees inferred using SSU rDNA of Myxosporea. The alignments (Alignment 1–3) are described in Material and Methods. Alignment 1: 2711 positions, 975 constant, 437 variable parsimony-uninformative, 1,300 parsimony-informative. Alignment 2: 1756 (64% of the original 2711 positions), 689 constant, 298 variable parsimony-uninformative, 769 parsimony-informative. Alignment 3: 1206 (44% of the original 2711 positions), 551 constant, 209 variable parsimony-uninformative, 446 parsimony-informative. The trees were reconstructed using PhyML 3.0 (GTR+G+I) [67], PAUP*4b10 (D.L. Swofford, 2001, PAUP*, Sunderland, MA: Sinauer Associates) with models selected using AIC in ModelTest3.7 (Alignment 1: GTR+G, Alignment 2: GTR+G+I, Alignment 3: GTR+G+I) [69], MrBayes3.1.2 (GTR+G+I) [68]. The maximum likelihood phylogeny (PhyML) tree robustness was assessed by the bootstrapping method with 500 replicates [41]. Bayesian support (posterior probability) for assessed (MrBayes) using two independent runs and four Markov chains and 40% steps discarded as a burn-in [68]. All trees were rooted using Buddenbrockia plumatellae sequence. The sequence taxa are on the right of the tree (for details see Table S4). (PDF) [file pone.0018871.s005.pdf]
